# Complete genome sequences of *Micrococcus luteus* isolated from the radioactive element-containing water in Fukushima Daiichi nuclear power station unit 2

**DOI:** 10.1128/mra.00769-25

**Published:** 2025-08-25

**Authors:** Yuma Dotsuta, Itsuki Taniguchi, Yasuhiro Gotoh, Tetsuya Hayashi, Ken Kurokawa, Tomoro Warashina, Akio Kanai, Toru Kitagaki

**Affiliations:** 1 Collaborative Laboratories for Advanced Decommissioning Science, Japan Atomic Energy Agency, Tokai, Japan; 2 Department of Bacteriology, Faculty of Medical Sciences, Kyushu University 12923, , Fukuoka, Japan; 3 Advanced Genomics Center, National Institute of Genetics 26359, Shizuoka, Japan; 4 Institute for Advanced Biosciences, Keio University 74060, Tsuruoka, Japan; University of Southern California, Los Angeles, California, USA

**Keywords:** bacteria, radiation, DNA, genome analysis, Fukushima Daiichi NPS, *Micrococcus luteus*, adaptive mutations

## Abstract

Four bacterial strains with yellow-colored colonies, which were isolated from the radioactive element-containing water in Fukushima Daiichi nuclear power station unit 2, were identified as *Micrococcus luteus*. Here, we present the complete genome sequences of these species assembled via a combination of short-read and long-read sequencing techniques.

## ANNOUNCEMENT

Highly radioactive water has accumulated in the torus room of Fukushima Daiichi Nuclear Power Station Unit 2 since the 2011 accident (1, 2). High chloride levels suggest remnants of initially injected seawater (3). A water sample collected on 30 June 2020 (1.55 × 10⁶ Bq/L β-ray, mainly cesium) contains a seawater-like bacterial community that survived under high radiation (4). Due to culturing difficulty, 100 µL of the sample was diluted in 10 mL of marine broth 2216 (Becton, Dickinson and Co. (BD), Franklin Lakes, NJ) and cultured by incubating at 30°C for a week. This step was repeated four times to reduce radiation. Subsequently, the sample was filtered through a 0.2 µm membrane filter (Corning) to trap non-radioactive microorganisms, which were then resuspended in the same liquid medium and cultured by incubating at 30°C on 1.5% marine broth 2216 agar plates. Four yellow colonies (1F2-TY1 to TY4) were isolated.

Each colony was cultured in the same liquid medium. High-molecular-weight DNA for Nanopore and short-read DNA for Illumina sequencing were extracted from each liquid medium using NucleoBond HMW DNA Kit and NucleoSpin Microbial DNA Kit (Macherey-Nagel, Düren, Germany), respectively. To obtain long-read sequences, sequence libraries were prepared with the Rapid Barcoding Kit SQK-RBK004 (Oxford Nanopore Technologies (ONT), Oxford, UK), without size selection. Libraries were loaded into an R9.4.1 flow cell (ONT) and run on a MinION Mk1C system (ONT). Raw reads were basecalled using the high-accuracy configuration of Guppy (v.6.5.7) (5), adapters were removed with Porechop (v.0.2.4) (6), and reads shorter than 20 kbp and low-quality reads (quality <10) were filtered using NanoFilt (v.2.8.0) (7) with the following flags: --quality 10 --length 20000. Corresponding paired-end 301 bp short reads were obtained from libraries prepared with the NEBNext Ultra II FS DNA Library Prep Kit for Illumina (New England Biolabs, Ipswich, MA), followed by consecutive sequencing with v.3 reagents on an Illumina MiSeq system (Illumina, San Diego, CA). Reads shorter than 25 bp and low-quality reads (quality <15) were filtered using Atria (v.3.2.2) (8). The long-read assembly and polishing were performed using the MicroPIPE (9) pipeline. In brief, trimmed long reads were assembled using Flye (v2.9-b1768) (10) and polished with the long reads by four iterations using Racon (v1.4.20) (11), followed by one iteration using Medaka (v1.4.3) (GitHub – https://github.com/nanoporetech/medaka). Output contigs were further refined with Illumina short reads using NextPolish (v1.3.1) (GitHub – https://github.com/Nextomics/NextPolish). Default parameters were used for all software unless otherwise specified. The genome assembly metrics for each isolate are listed in Table 1. Annotations were performed using the DFAST (v.1.2.17) (12). The chromosomes of the four strains are circular, and each strain contains a linear plasmid, identified by the presence of a gene homologous to repA and a region similar to plasmid sequences of *M. luteus* (13) with NCBI BLAST (14). While the ANI values analyzed by PyANI (v.0.2.12) (15) between 1F2-TY1 and 1F2-TY2 were 100%, those among 1F2-TY1/1F2-TY2, 1F2-TY3, and 1F2-TY4 were 97.4–97.9%, and those of each strain against the type strain of *M. luteus* (NCTC2665, DDBJ accession no. CP001628) were 96.7–97.0%.

**TABLE 1:** Summary of complete genome sequences of four *Micrococcus luteus* strains isolated from the radioactive contaminated water in the torus room of Fukushima Daiichi Nuclear Power Station Unit 2

Strain	No. of Illumina reads	No. of Nanopore reads	Nanopore read N_50_ (bp)	Chromosome Plasmid	Length (bp)	Coverage (×)	G + C content (%)	No. of coding sequences	DDBJ accession no.	Illumina SRA accession no.	Nanopore SRA accession no.
1F2-TY1	627,952	84,788	17,333	Chromosome	2,488,561	88	73.12	2,259	AP041098	DRR595938	DRR595945
				Plasmid	99,801	347	69.46	116	AP041099		
1F2-TY2	655,438	42,884	24,862	Chromosome	2,486,336	115	73.12	2,258	AP041100	DRR595939	DRR595946
				Plasmid	99,401	512	69.40	116	AP041101		
1F2-TY3	848,860	23,192	18,298	Chromosome	2,474,352	34	73.05	2,277	AP041102	DRR595940	DRR595947
				Plasmid	95,098	164	69.77	107	AP041103		
1F2-TY4	714,710	56,357	22,613	Chromosome	2,439,246	127	73.1	2,237	AP041104	DRR595941	DRR595948
				Plasmid	128,267	393	69.42	146	AP041105		

## Data Availability

The final complete genome sequences and the sequences of Illumina reads, Nanopore reads, and assemblies have been deposited in DDBJ under the accession numbers listed in Table 1.

## References

[1] TEPCO. 2013. Investigation of Unit 2 Torus Room at Fukushima Daiichi Nuclear Power Station. Available from: https://www.tepco.co.jp/en/nu/fukushima-np/handouts/2013/images/handouts_130412_08-e.pdf. Retrieved 18 Aug 2025.

[2] TEPCO. 2012. Investigation of the torus room in the basement of reactor building 2. Available from: https://www.tepco.co.jp/nu/fukushima-np/images/handouts_120419_01-j.pdf. Retrieved 22 Apr 2024.

[3] TEPCO. 2019. Analysis results of alpha nuclides in the water retained in the reactor building. Available from: https://www.meti.go.jp/earthquake/nuclear/decommissioning/committee/osensuitaisakuteam/2019/06/3-1-2.pdf. Retrieved 22 Apr 2024.

[4] Warashina T, Sato A, Hinai H, Shaikhutdinov N, Shagimardanova E, Mori H, Tamaki S, Saito M, Sanada Y, Sasaki Y, Shimada K, Dotsuta Y, Kitagaki T, Maruyama S, Gusev O, Narumi I, Kurokawa K, Morita T, Ebisuzaki T, Nishimura A, Koma Y, Kanai A. 2024. Microbiome analysis of the restricted bacteria in radioactive element-containing water at the Fukushima Daiichi Nuclear Power Station. Appl Environ Microbiol 90:e0211323. doi:10.1128/aem.02113-23 38470121 PMC11022576

[5] Wick RR, Judd LM, Holt KE. 2019. Performance of neural network basecalling tools for Oxford Nanopore sequencing. Genome Biol 20:129. doi:10.1186/s13059-019-1727-y 31234903 PMC6591954

[6] Wick RR, Judd LM, Gorrie CL, Holt KE. 2017. Completing bacterial genome assemblies with multiplex MinION sequencing. Microb Genom 3:e000132. doi:10.1099/mgen.0.000132 29177090 PMC5695209

[7] De Coster W, D’Hert S, Schultz DT, Cruts M, Van Broeckhoven C. 2018. NanoPack: visualizing and processing long-read sequencing data. Bioinformatics 34:2666–2669. doi:10.1093/bioinformatics/bty149 29547981 PMC6061794

[8] Chuan J, Zhou A, Hale LR, He M, Li X. 2021. Atria: an ultra-fast and accurate trimmer for adapter and quality trimming. GigaByte 2021:gigabyte31. doi:10.46471/gigabyte.31 36967729 PMC10038132

[9] Murigneux V, Roberts LW, Forde BM, Phan MD, Nhu NTK, Irwin AD, Harris PNA, Paterson DL, Schembri MA, Whiley DM, Beatson SA. 2021. MicroPIPE: validating an end-to-end workflow for high-quality complete bacterial genome construction. BMC Genomics 22:474. doi:10.1186/s12864-021-07767-z 34172000 PMC8235852

[10] Kolmogorov M, Yuan J, Lin Y, Pevzner PA. 2019. Assembly of long, error-prone reads using repeat graphs. Nat Biotechnol 37:540–546. doi:10.1038/s41587-019-0072-8 30936562

[11] Vaser R, Sović I, Nagarajan N, Šikić M. 2017. Fast and accurate de novo genome assembly from long uncorrected reads. Genome Res 27:737–746. doi:10.1101/gr.214270.116 28100585 PMC5411768

[12] Tanizawa Y, Fujisawa T, Nakamura Y. 2018. DFAST: a flexible prokaryotic genome annotation pipeline for faster genome publication. Bioinformatics 34:1037–1039. doi:10.1093/bioinformatics/btx713 29106469 PMC5860143

[13] Wong CACM, Lai GKK, Griffin SDJ, Leung FCC. 2022. Complete genome sequence of Micrococcus luteus strain CW.Ay, isolated from indoor air in a Hong Kong school. Microbiol Resour Announc 11:e01194-21. doi:10.1128/mra.01194-21 35175116 PMC8852316

[14] Priyam A, Woodcroft BJ, Rai V, Moghul I, Munagala A, Ter F, Chowdhary H, Pieniak I, Maynard LJ, Gibbins MA, Moon H, Davis-Richardson A, Uludag M, Watson-Haigh NS, Challis R, Nakamura H, Favreau E, Gómez EA, Pluskal T, Leonard G, Rumpf W, Wurm Y. 2019. Sequenceserver: a modern graphical user interface for custom BLAST databases. Mol Biol Evol 36:2922–2924. doi:10.1093/molbev/msz185 31411700 PMC6878946

[15] Pritchard L, Glover RH, Humphris S, Elphinstone JG, Toth IK. 2016. Genomics and taxonomy in diagnostics for food security: soft-rotting enterobacterial plant pathogens. Anal Methods 8:12–24. doi:10.1039/C5AY02550H

